# Association between leukocyte-derived inflammatory biomarkers and diabetic retinopathy progression in patients with type 2 diabetes mellitus

**DOI:** 10.3389/fendo.2026.1926059

**Published:** 2026-09-24

**Authors:** Tong Wu, Ying Xie

**Affiliations:** 1 Department of General Practice, Shanxi Provincial People’s Hospital Affiliated to Shanxi Medical University, Taiyuan, Shanxi, China; 2 Department of Ophthalmology, Shanxi Provincial People’s Hospital Affiliated to Shanxi Medical University, Taiyuan, Shanxi, China

**Keywords:** biomarkers, diabetic retinopathy, lymphocytes, neutrophils, platelet-to-lymphocyte ratio, progression

## Abstract

**Objective:**

To characterize changes in leukocyte-based inflammatory biomarkers in patients with diabetic retinopathy (DR) progression, using the DR non-progression group as a control, and to evaluate the association between these biomarkers and DR progression.

**Methods:**

A total of 70 patients with T2DM were included in the study, comprising 40 in the DR progression group and 30 in the DR non-progression group. Based on scanning laser ophthalmoscopy findings, participants were categorized into the DR progression group or the DR non-progression group. Changes in leukocyte-based inflammatory biomarkers between the two time points were compared within each group, and the associations between changes in biomarkers and DR progression were then analyzed.

**Results:**

No significant differences in baseline characteristics were observed between the two groups. In the DR progression group, neutrophil (NE) and lymphocyte (LYM) counts were significantly lower at the time of progression than at baseline, whereas the platelet-to-lymphocyte ratio (PLR) was significantly higher. No significant changes were observed in the remaining inflammatory biomarkers. In the DR non-progression group, none of the evaluated inflammatory biomarkers demonstrated significant differences between the two assessment time points. Correlation analysis demonstrated that DR progression was negatively associated with changes in NE count, whereas no significant associations were identified between DR progression and changes in LYM counts or PLR.

**Conclusion:**

Among the leukocyte-based inflammatory biomarkers evaluated, a decrease in NE count was significantly associated with DR progression. Although LYM and PLR showed significant within-group changes over time, their correlation with progression status was not statistically significant, suggesting that their roles require further validation.

## 1 Introduction

With improvements in living standards and the increasing adoption of unhealthy lifestyle behaviors, the prevalence of diabetes mellitus (DM) in China has risen steadily. Epidemiological studies have estimated that approximately 116 million individuals in China were living with DM in 2019, and this number is projected to increase to 147 million by 2030 (1). In addition, the incidence of DM among younger populations has increased substantially, resulting in a growing burden of diabetes-related complications during the most economically productive years of life (2). Among these complications, diabetic retinopathy (DR) is one of the most common and remains a leading cause of vision loss and blindness worldwide. A previous survey reported a DR prevalence of 34.6% among individuals with DM (3).

Despite extensive research on DR, clinical management still relies on specialized retinal imaging, whose limited accessibility in primary care settings often delays diagnosis and increases the risk of irreversible vision loss. There is an urgent need for cost-effective, readily available alternatives suitable for routine use. Leukocyte-based inflammatory biomarkers, including neutrophil-to-lymphocyte ratio (NLR), platelet-to-lymphocyte ratio (PLR), and monocyte-to-lymphocyte ratio (MLR), may serve as practical surrogate indicators for DR progression, potentially addressing current diagnostic gaps, particularly in resource-limited regions.

Inflammation is widely recognized as a key contributor to the development and progression of DR. The underlying mechanisms are complex and involve interactions among multiple molecular and cellular pathways. Chronic hyperglycemia induces leukocyte activation and infiltration and promotes inflammatory responses in retinal microvascular endothelial cells, pericytes, and neurons (4). These processes stimulate the production of pro-inflammatory cytokines and chemokines, leading to the recruitment and activation of immune cells, including macrophages and microglia, thereby establishing a pro-inflammatory microenvironment within the retina (5). Persistent inflammation further compromises the integrity of the blood-retinal barrier, increases vascular permeability, and contributes to retinal edema and hard exudate formation. Furthermore, inflammatory signaling promotes vascular endothelial growth factor (VEGF) expression through pathways involving transcription factors such as nuclear factor-kappa B (NF-κB), thereby facilitating pathological retinal neovascularization. These newly formed vessels are structurally fragile and prone to rupture and hemorrhage, resulting in further retinal damage (6, 7).

Accumulating evidence indicates that leukocyte extravasation involving neutrophils (NE), lymphocytes (LYM), and monocytes (Mo) is a central feature of the inflammatory processes associated with DR. Studies have suggested that neutrophils and neutrophil extracellular traps may contribute to the pathogenesis of DR, potentially through mechanisms involving Toll-like receptors and lysyl oxidase (8, 9). Furthermore, a cross-sectional study reported that reduced peripheral blood Mo counts were significantly associated with the onset of DR (10). Elevated monocyte–platelet aggregate levels have also been observed in individuals with proliferative diabetic retinopathy (PDR) (11). However, findings regarding peripheral blood leukocyte counts in DR remain inconsistent. To address this, recent investigations have integrated routine hematological parameters to develop novel inflammatory indices, including the NLR, PLR, MLR, systemic inflammatory response index (SIRI), systemic immune-inflammation index (SII), and aggregate index of systemic inflammation (AISI) (12). These composite indices reflect the combined contributions of platelets, neutrophils, lymphocytes, and monocytes to systemic inflammatory responses and may provide more accurate measures of inflammatory status in individuals with DR. Previous studies have identified NLR, PLR, MLR, and SII as potential biomarkers to guide therapeutic decision-making in diabetic macular edema (DME) (13). Additional studies have demonstrated that NLR, PLR, and SII are each associated with the presence and progression of DR, with elevated levels of NLR and PLR reflecting increased disease risk and greater severity (14). Beyond their established roles in DR, the aforementioned composite inflammatory indicators have also been closely associated with the prognosis of various other diseases, including in patients with arthritis and comorbid hypertension, as well as in elderly patients with sepsis (15, 16).

The present study aimed to evaluate longitudinal changes in leukocyte-based inflammatory biomarkers, including NE, LYM, Mo, and their derived indices (NLR, PLR, MLR, SIRI, SII, and AISI), in the same patient with DR at two time points. By characterizing changes in these biomarkers, their associations with DR progression and potential clinical utility were assessed. The findings may provide evidence supporting the use of leucocyte-based inflammatory biomarkers for the identification and dynamic monitoring of DR progression. Furthermore, the results may contribute to the development of cost-effective monitoring strategies, particularly in primary healthcare settings where access to specialized ophthalmic resources remains limited.

## 2 Patients and methods

### 2.1 Study participants

A total of 70 patients with type 2 diabetes mellitus (T2DM) who met the predefined inclusion and exclusion criteria were enrolled in this study. All participants had been hospitalized on two occasions in the Department of Endocrinology of Shanxi Provincial People’s Hospital between October 2022 and December 2024. All patients in this cohort were admitted on both occasions for routinely scheduled follow-up assessments as part of their standard diabetes care, rather than for acute complications or emergent indications. Fundus examinations were performed by two senior retinal specialists. The severity of DR was graded according to the International Clinical Diabetic Retinopathy Disease Severity Scale proposed by the International Council of Ophthalmology in 2002 (17). Based on changes in DR severity between the two examinations, participants were categorized into either the DR progression group (*n* = 40) or the DR non-progression group (*n* = 30). The flowchart is shown in **Figure 1**.

**Figure 1:**
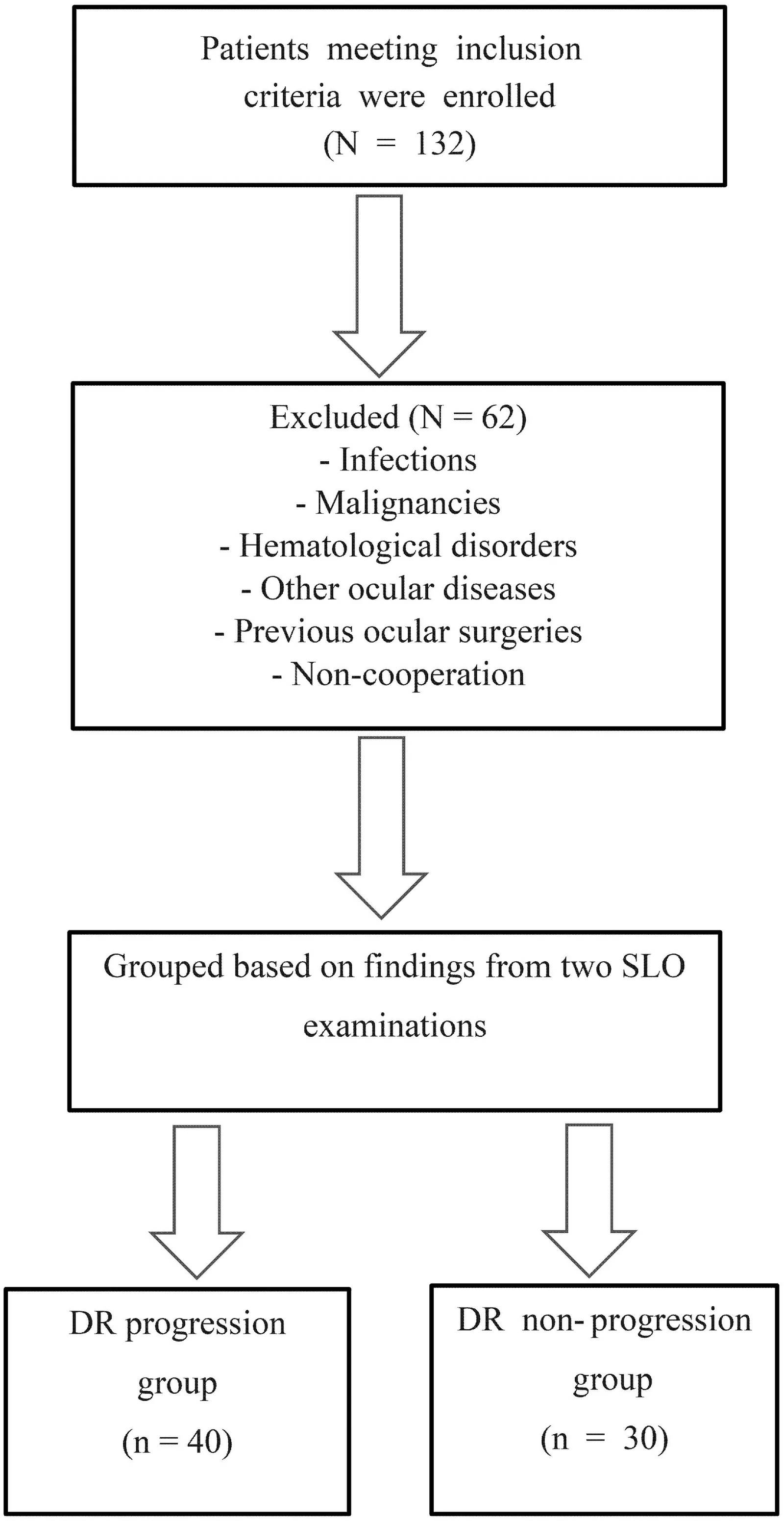
Participant flow diagram.

Inclusion criteria: 1) Diagnosis of T2DM according to established diagnostic criteria (18); 2) Age between 18 and 80 years; 3) Refractive error ≤ ± 6.00 diopters (D); 4) Intraocular pressure between 10 and 21 mmHg; 5) Completion of scanning laser ophthalmoscopy (SLO) examinations with images of sufficient quality for DR grading.

Exclusion criteria: 1) Type 1 diabetes mellitus (T1DM), gestational DM, or other specific types of diabetes; 2) Conditions known to influence inflammatory biomarkers, including infectious diseases, malignancies, or autoimmune disorders; 3) Factors affecting complete blood count (CBC) parameters, including hematological disorders, blood transfusion, or the use of medications that alter blood cell counts; 4) Coexisting ocular diseases, including glaucoma, age-related macular degeneration, retinal vein occlusion, or significant cataract; 5) Previous ocular interventions, including retinal photocoagulation, intravitreal injection, or vitrectomy; 6) Psychiatric disorders or other conditions that precluded completion of fundus examinations, or loss to follow-up.

Grouping criteria: 1) The DR progression group comprised participants who exhibited progression on serial SLO examinations, including the following transitions: from no diabetic retinopathy (NDR) to non-proliferative diabetic retinopathy (NPDR), of any severity; from NDR to PDR; from a lower to a higher NPDR severity grade; or from NPDR to PDR; 2) The DR non-progression group comprised participants whose DR grade remained unchanged between the two SLO examinations; 3) In cases of disagreement between the two retinal specialists regarding DR classification, a third specialist independently reviewed the images, and the final grade was determined by majority agreement between at least two of the three specialists; and 4) For participants with asymmetric DR severity between the two eyes, classification was determined according to the eye with the more advanced stage of disease.

This study was conducted in accordance with the principles of the World Medical Association Declaration of Helsinki and was approved by the Ethics Committee of Shanxi Provincial People’s Hospital. Written informed consent was obtained from all participants prior to enrollment.

### 2.2 Study methods

#### 2.2.1 Collection of basic data

Demographic and clinical data, including age, sex, height, and weight, were extracted from the medical records at both time points. The interval between the two hospitalizations was recorded, along with vital signs, including body temperature, pulse rate, respiratory rate, and blood pressure. Body mass index (BMI) was calculated using the following formula: BMI = weight (kg)/[height (m)]².

#### 2.2.2 Collection of laboratory data

Laboratory data obtained at both time points included LYM count, NE count, Mo count, platelet (PLT) count, fasting plasma glucose, hemoglobin A1c, and other routine laboratory parameters.

Composite inflammatory indices were calculated as follows:

NLR = NE/LYM

PLR = PLT/LYM

MLR = Mo/LYM

SIRI = NE Count × Mo Count/LYM Count

SII = PLT Count × NE Count/LYM Count

AISI = NE × PLT × (Mo/LYM)

#### 2.2.3 Evaluation indices

Levels of LYM, NE, Mo, PLT, NLR, PLR, MLR, SIRI, SII, and AISI were compared between the DR progression group and the DR non-progression group at each time point. In addition, the associations between DR progression and longitudinal changes in these biomarkers were evaluated.

### 2.3 Statistical analysis

Statistical analyses were performed using SPSS version 27.0. The normality of continuous variables was assessed using the Shapiro–Wilk test. Normally distributed variables were expressed as mean ± standard deviation ( $\bar{X}$ ± *SD*). Between-group comparisons were conducted using the independent-samples t-test, whereas within-group comparisons were performed using the paired-samples t-test. Non-normally distributed variables were presented as median (interquartile range [IQR]). Between-group comparisons were conducted using the Mann–Whitney U test, and within-group comparisons were performed using the Wilcoxon signed-rank test. Categorical variables were expressed as frequencies and percentages, and between-group differences were assessed using the chi-square test or Fisher’s exact test, as appropriate. Associations between DR progression and changes in inflammatory biomarkers were evaluated using point-biserial correlation analysis. All statistical tests were two-sided. Given the exploratory nature of the study, p < 0.05 was used as a nominal threshold for statistical significance, and no adjustment for multiple comparisons was performed. Therefore, statistically significant findings should be regarded as exploratory, intended to generate research hypotheses rather than provide confirmatory evidence.

## 3 Results

### 3.1 Comparison of general data

Comparisons of sex, age, height, weight, BMI, and vital signs at the first assessment revealed no significant differences between the DR progression group and the DR non-progression group (all *p* > 0.05) (**Table 1**).

**Table 1:** Baseline demographic and clinical characteristics of the DR progression and DR non-progression groups at the first assessment.

		DR progression group (*n* = 40)	DR non-progression group (*n* = 30)	Statistic	*p*-value
Sex	Male	29 (72.5%)	19 (63.3%)	0.668	0.414
	Female	11 (27.5%)	11 (36.7%)		
Age (years)	Median	56.50 (11)	58.50 (16)	-0.707	0.480
Height (cm)	Mean ± SD	168.30 ± 7.67	167.87 ± 9.77	0.208	0.836
Weight (kg)	Median	69 (16)	68 (20)	-0.612	0.540
BMI (kg/m2)	Mean ± SD	25.09 ± 2.78	24.80 ± 3.81	0.374	0.709
Heart rate (beats/min)	Mean ± SD	70.43 ± 7.42	71.73 ± 6.65	-0.085	0.923
Respiratory rate (breaths/min)	Median	19 (2)	20 (2)	-0.670	0.469
Body temperature (°C)	Median	36.4 (0.4)	36.5 (0.3)	-0.156	0.853
Systolic blood pressure (mmHg)	Median	120 (15)	121 (12)	-0.313	0.745
Diastolic blood pressure (mmHg)	Median	78 (8)	82 (9)	-0.492	0.658

At the second assessment, no significant differences were observed between the two groups in height, weight, BMI, or vital signs at the second hospitalization (all *p* > 0.05) (**Table 2**).

**Table 2:** Demographic and clinical characteristics of the DR progression and DR non-progression groups at the second assessment.

		DR progression group (*n* = 40)	DR non-progression group (*n* = 30)	Statistic	*p*-value
Height (cm)	Median	168.33 ± 7.79	167.57 ± 9.53	0.366	0.715
Weight (kg)	Mean ± SD	70.83 ± 11.45	68.73 ± 14.18	0.683	0.497
BMI(kg/m2)	Median	24.89 ± 2.85	24.31 ± 3.48	0.773	0.442
Heart rate (beats/min)	Mean ± SD	70.56 ± 6.98	71.05 ± 7.48	-0.276	0.745
Respiratory rate (breaths/min)	Mean ± SD	18(3)	19(2)	-0.692	0.357
Body temperature (°C)	Median	36.5(0.4)	36.6(0.3)	-0.226	0.552
Systolic blood pressure (mmHg)	Median	119(15)	121(12)	-0.509	0.741
Diastolic blood pressure (mmHg)	Median	78(7)	80(9)	-0.492	0.667

The time interval between the two hospitalizations was also comparable between the two groups, with no significant difference observed (*p* > 0.05) (**Table 3**).

**Table 3:** Comparison of the time interval between the two hospitalizations in both groups.

	DR progression group (*n* = 40)		DR non-progression group (*n* = 30)	Statistic	*p*-value
Time interval (months)	Mean ± SD	14.93 ± 5.25	14.97 ± 4.96	-0.034	0.973

### 3.2 Comparison of clinical diagnostic stages

No significant difference in DR severity grade at baseline was observed between the DR progression group and the DR non-progression group (*p* > 0.05) (**Table 4**).

**Table 4:** Comparison of clinical diabetic retinopathy stages at the first hospitalization in both groups.

Clinical diagnostic staging	DR progression group (*n* = 40)	DR non-progression group (*n* = 30)	Statistic	*p*-value
NDR	13	16	6.768	0.079
Mild NPDR	20	14		
Moderate NPDR	5	0		
Severe NPDR	1	0		
PDR	1	0		

### 3.3 Distribution of DR stage transitions in the progression group

Of the 40 patients in the progression group, the distribution of DR stage transitions was as follows: 8 patients progressed from NDR to mild NPDR, 4 from NDR to moderate NPDR, 1 from NDR to severe NPDR, 17 from mild NPDR to moderate NPDR, 3 from mild NPDR to severe NPDR, 5 from moderate NPDR to severe NPDR, 1 from severe NPDR to PDR, and 1 patient with pre-existing PDR showed worsening of the disease.

### 3.4 Changes in leukocyte-based inflammatory biomarkers in the DR progression group

In the DR progression group, NE and LYM counts were significantly lower at the second assessment than at baseline (both *p* < 0.05). PLR was significantly higher at the second assessment than at baseline (*p* < 0.05). No significant differences were observed in Mo count, PLT count, NLR, MLR, SIRI, SII, or AISI between the two time points (all *p* > 0.05) (**Table 5**).

**Table 5:** Comparison of leukocyte-based inflammatory biomarkers before and after diabetic retinopathy progression in the DR progression group.

		Before DR progression	After DR progression	Statistic	*p*-value
NE (*10^9^/L)	Mean ± SD	3.86 ± 1.02	3.46 ± 0.85	2.648	0.012
LYM(*10^9^/L)	Mean ± SD	1.84 ± 0.59	1.73 ± 0.60	2.640	0.012
Mo (*10^9^/L)	Mean ± SD	0.45 ± 0.12	0.41 ± 0.13	1.883	0.067
PLT (*10^9^/L)	Mean ± SD	205.5 ± 53.1	209.6 ± 49.1	-0.633	0.531
NLR	Mean ± SD	2.27 ± 0.85	2.23 ± 0.92	0.364	0.718
PLR	Mean ± SD	120.8 ± 40.5	135.1 ± 53.0	-2.497	0.017
MLR	Mean ± SD	0.26 ± 0.076	0.26 ± 0.105	-0.003	0.998
SII	Mean ± SD	466.0 ± 199.5	473.0 ± 253.1	-0.221	0.826
SIRI	Mean ± SD	1.00 ± 0.41	0.91 ± 0.48	1.191	0.241
AISI	Median	188(142)	150(135)	-1.452	0.150

### 3.5 Changes in leukocyte-based inflammatory biomarkers in the DR non-progression group

In the DR non-progression group, no significant differences were observed in NE count, LYM count, Mo count, PLT count, NLR, PLR, MLR, SIRI, SII, or AISI between the two assessments (all *p* > 0.05) (**Table 6**).

**Table 6:** Comparison of leukocyte-based inflammatory biomarkers between the two hospitalizations in the DR non-progression group.

		First measurement of the non-progression	Second measurement of the non-progression groupgroup	Statistic	*p*-value
NE (*10^9^/L)	Mean ± SD	3.27 ± 0.79	3.31 ± 0.79	-0.324	0.748
LYM(*10^9^/L)	Mean ± SD	2.10 ± 0.58	2.06 ± 0.64	0.529	0.601
Mo (*10^9^/L)	Mean ± SD	0.46 ± 0.13	0.44 ± 0.14	1.056	0.594
PLT (*10^9^/L)	Mean ± SD	221.5 ± 55.9	218.4 ± 53.5	0.538	0.299
NLR	Mean ± SD	1.67 ± 0.61	1.76 ± 0.68	-0.870	0.391
PLR	Mean ± SD	112.2 ± 38.7	113.5 ± 40.8	-0.248	0.806
MLR	Mean ± SD	0.23 ± 0.084	0.22 ± 0.075	0.842	0.550
SII	Mean ± SD	363.4 ± 139.2	377.8 ± 157.3	-0.605	0.407
SIRI	Mean ± SD	0.77 ± 0.31	0.75 ± 0.32	0.280	0.782
AISI	Median	173(127)	168(89)	-0.586	0.570

### 3.6 Correlation between changes in NE, LYM, and PLR and DR progression

Point-biserial correlation analysis demonstrated a significant negative correlation between DR progression and change in NE count (*r* = -0.243, 95% CI: -0.452 to -0.008, *p* < 0.05), indicating that a greater reduction in NE count was associated with a higher likelihood of DR progression. No significant correlations were identified between DR progression and changes in LYM count or PLR (both *p* > 0.05) (**Table 7**).

**Table 7:** Correlation analysis of changes in neutrophil, lymphocyte, and platelet-to-lymphocyte ratio values with DR progression.

	*R vaule*	*P vaule*
Changes in NE values	-0.243	0.043
Changes in LYM values	-0.101	0.406
Changes in PLR values	0.193	0.109

## 4 Discussion

In the present study, the female-to-male ratio among the enrolled participants was 1:2.18, indicating a substantially higher proportion of males than females. Participant age ranged from 24 to 80 years, with a mean age of 56.27 years. The mean BMI was 24.97 kg/m², which falls within the overweight range. Previous studies have established a closer association between male sex and the prevalence of DR (19), a demographic trend that is reflected in the predominance of male participants enrolled in our study. In the present study, the majority of enrolled patients were over 50 years of age, which is consistent with the known epidemiology of DR, as advanced age is a well-established risk factor for its development (20). Furthermore, a positive linear association has been reported between BMI and DR prevalence, with higher BMI associated with an increased risk of DR. BMI has also been identified as an independent predictor of DR risk among older adults (21). This relationship may be partially attributable to altered adipokine profiles in obesity, characterized by increased pro-inflammatory and reduced anti-inflammatory adipokine levels (22). In addition to multidisciplinary management involving endocrinologists, ophthalmologists, and primary care physicians, lifestyle modifications, including a healthy diet, regular moderate-intensity physical activity, and weight management, remain important strategies for reducing DR risk and delaying progression.

Despite advances in DR management, the disease remains a leading cause of visual impairment worldwide, affecting over 2.9 million adults aged ≥50 years in 2020, with projections reaching 160.5 million by 2045 (23, 24). Resource constraints in underserved regions often delay timely diagnosis, underscoring the need for accessible biomarkers to monitor disease progression. Composite inflammatory indices derived from routine blood counts—including NLR, PLR, MLR, SII, SIRI, and AISI—have emerged as promising measures of systemic inflammation (25). These biomarkers have shown potential utility in the diagnosis and prognosis of various chronic diseases (26–28), and may offer practical tools for DR monitoring, particularly in resource-limited settings.

In one large-scale study, Wan et al. compared hematologic parameters between 2,709 individuals without DR and 512 individuals with DR (10). The authors reported no significant differences in NE or PLT counts in early DR, whereas Mo counts were significantly lower in the DR group. Huang et al. reported that MLR may have value in distinguishing individuals with T2DM and DR from those with T2DM without DR (29). However, other studies have indicated that MLR has limited diagnostic utility for DR and PDR, whereas NLR and PLR may be more strongly associated with disease presence and severity (30). Zeng et al. reported higher NLR and PLR values in individuals with DR than in those without DR, and multivariable logistic regression identified PLR as an independent predictor of DR (31). Several investigators have also explored cutoff values for NLR and PLR to differentiate individuals with DR from those without and to stratify disease severity (32).

More recently, attention has shifted toward the clinical relevance of SII and SIRI in DR. Eybeli et al. reported that both SII and diabetes duration were associated with the risk of DME among individuals with NPDR (33). Consistent with these findings, Wang et al. demonstrated significantly higher SII values in individuals with DME than in those without DME (34). Furthermore, elevated SII and SIRI levels have been independently associated with DR and may serve as potential indicators of DR development in individuals with T2DM. Collectively, these findings suggest that leukocyte-based inflammatory biomarkers may have value in the early identification, risk stratification, and prognostic assessment of DR, although further studies are needed to establish their clinical applicability and standardized diagnostic thresholds.

The present study demonstrated that, among patients with DR progression, NE and LYM counts decreased significantly, whereas PLR increased significantly between assessments. In contrast, no significant changes in these biomarkers were observed among patients without DR progression. These within-group findings suggest that these biomarkers may exhibit dynamic changes accompanying DR progression. However, correlation analysis revealed that only the change in NE count was significantly associated with DR progression, indicating that greater reductions in NE counts were associated with a higher likelihood of DR progression. Although the observed change in NE was significant, the effect size was relatively modest, and its clinical relevance remains to be determined. In contrast, the changes in LYM and PLR did not reach statistical significance in the correlation analysis, suggesting that their alterations may reflect binary state changes rather than a linear relationship with the degree of progression. The finding regarding NE is consistent with previous studies highlighting the involvement of neutrophils in DR pathogenesis and progression. Although the underlying mechanisms remain incompletely understood, previous studies have suggested that the observed association may be related to the release of inflammatory mediators, such as elastase, by neutrophils (35). The roles of LYM and PLR remain exploratory and require further validation in larger cohorts.

The role of inflammatory mediators in the pathogenesis of DR has been extensively investigated, yet the literature remains inconclusive. These discrepancies may be attributed to differences in study design, population characteristics, and methodological approaches. By employing a longitudinal within-patient design, the present study offers a complementary perspective. The consistencies and inconsistencies between our findings and previous reports reflect the complexity of the inflammatory pathways involved in DR and underscore the need for standardized, large-scale prospective studies to further elucidate these mechanisms.

In conclusion, by longitudinally evaluating changes in leukocyte-based inflammatory biomarkers within the same cohort, this study provides evidence linking systemic inflammation to DR progression. Several biomarkers demonstrated dynamic changes during disease worsening and may be candidate markers for further investigation. If validated in larger prospective studies, these biomarkers may contribute to the assessment and prognostic evaluation of DR. Furthermore, given that CBC testing is widely available, technically straightforward, and cost-effective, these indices are well-suited for DR monitoring, particularly in primary healthcare and resource-limited settings.

### 4.1 Limitations

Several limitations should be acknowledged, including the small sample size, single-center design, and lack of subgroup analysis for patients who progressed to severe DR, which may limit statistical power and generalizability.

Other time-varying factors, including glycemic control (as reflected by HbA1c and FPG levels), may also have changed over the study period and could potentially affect both DR progression and the levels of inflammatory biomarkers. Although the within-patient longitudinal design reduces confounding from stable individual characteristics, it cannot eliminate confounding from time-varying treatment changes or fluctuations in metabolic control. Residual confounding related to medication changes and glycemic variability therefore cannot be excluded, but the effects of glucose-lowering agents on inflammatory biomarkers remain inconclusive (36, 37). In addition, detailed data on glucose-lowering medications were not collected in this study, and treatment adjustments during follow-up may have influenced inflammatory biomarker levels.

We also acknowledge that although our within-patient longitudinal paired analysis minimized inter-individual variability to characterize the inflammatory dynamics accompanying DR progression, unadjusted crude change score comparisons between groups without adequate covariate adjustment could be susceptible to confounding by regression to the mean and residual confounding. Our sample size was insufficient for robust adjusted between-group comparisons of biomarker changes.

Further investigation into the inflammatory mechanisms underlying DR and longitudinal changes in these biomarkers may provide valuable insights into DR pathophysiology, facilitate the development of accessible monitoring strategies, and support earlier identification of at-risk individuals. However, given the above limitations, larger multicenter studies with more diverse populations, detailed longitudinal treatment records, and comprehensive metabolic data are warranted to validate our findings and to strengthen causal inference linking leukocyte-derived biomarker dynamics to DR progression.

## 5 Conclusion

This exploratory study found that, among the leukocyte-based inflammatory biomarkers evaluated, a reduction in NE count was significantly associated with DR progression, and this finding warrants further investigation. Although LYM and PLR exhibited significant within-group changes over time, their correlation with progression status did not reach statistical significance, indicating that their roles remain preliminary and require further validation. It should be noted that the current study does not establish the diagnostic or predictive performance of these biomarkers; therefore, these findings should be considered preliminary and hypothesis-generating, requiring confirmation in larger prospective cohorts before any clinical application can be considered.

## Data Availability

The raw data supporting the conclusions of this article will be made available by the authors, without undue reservation.

## References

[1] Saeedi P Petersohn I Salpea P Malanda B Karuranga S Unwin N . Global and regional diabetes prevalence estimates for 2019 and projections for 2030 and 2045: Results from the International Diabetes Federation Diabetes Atlas. Diabetes Res Clin Pract. (2019) 157:107843. doi: 10.1016/j.diabres.2019.107843 31518657

[2] Luk AOY . Changing landscape of diabetes in Asia–What are the unmet needs? J Diabetes Invest. (2024) 15:402–9. doi: 10.1111/jdi.14150 38265148 PMC10981145

[3] Yau JWY Rogers SL Kawasaki R Lamoureux EL Kowalski JW Bek T . Global prevalence and major risk factors of diabetic retinopathy. Diabetes Care. (2012) 35:556–64. doi: 10.2337/dc11-1909 22301125 PMC3322721

[4] Hussain A Ashique S Afzal O Altamimi MA Malik A Kumar S . A correlation between oxidative stress and diabetic retinopathy: An updated review. Exp Eye Res. (2023) 236:109650. doi: 10.1016/j.exer.2023.109650 37734426

[5] Zhao B Zhao Y Sun X . Mechanism and therapeutic targets of circulating immune cells in diabetic retinopathy. Pharmacol Res. (2024) 210:107505. doi: 10.1016/j.phrs.2024.107505 39547465

[6] Wei L Sun X Fan C Wei L Sun X Fan C . The pathophysiological mechanisms underlying diabetic retinopathy. Front Cell Dev Biol. (2022) 10:963615. doi: 10.3389/fcell.2022.963615 36111346 PMC9468825

[7] Boneva SK Wolf J Jung M Prinz G Chui TYP Jauch J . The multifaceted role of vitreous hyalocytes: Orchestrating inflammation, angiomodulation and erythrophagocytosis in proliferative diabetic retinopathy. J Neuroinflamm. (2024) 21:297. doi: 10.1186/s12974-024-03291-5 39543723 PMC11566480

[8] Wu S Chen Y Jin X Yu J Chen X Wan T . Toll like receptors promote high glucose-induced vascular endothelial cell dysfunction by regulating neutrophil extracellular traps formation. Inflammation. (2025) 48:3542–59. doi: 10.1007/s10753-025-02283-8 40087251 PMC12596294

[9] Agarwal M Chandrakumar S Tierno IS Lessieur EM Bollinger ZR Kern TS . Lysyl oxidase promotes actin-dependent neutrophil activation and cytotoxicity in diabetes: Implications for diabetic retinopathy. Biorxiv. (2025). doi: 10.1101/2025.05.02.651525 PMC12716605 41187296

[10] Wan H Cai Y Wang Y Fang S Chen C Chen Y . The unique association between the level of peripheral blood monocytes and the prevalence of diabetic retinopathy: a cross-sectional study. J Transl Med. (2020) 18:1–9. doi: 10.1186/s12967-020-02422-9 32571335 PMC7310136

[11] Kaplar M Kappelmayer J Veszpremi A Szabo K Udvardy M . The possible association of *in vivo* leukocyte-platelet heterophilic aggregate formation and the development of diabetic angiopathy. Platelets. (2001) 12:419–22. doi: 10.1080/09537100120078368 11674859

[12] Gu W Wang M Li Z Xu T . The association between peripheral blood inflammatory markers and anti-VEGF treatment response in patients with type 2 diabetic macular edema. Front Med. (2025) 12:1653753. doi: 10.3389/fmed.2025.1653753 41179902 PMC12571745

[13] Harley O Amelia YS Gustianty E Soetedjo NN Kartasasmita AS . Exploring leukocyte differential count ratio profiles as inflammatory biomarkers in diabetic retinopathy: a systematic review and meta-analysis. BMC Ophthalmol. (2025) 25:265. doi: 10.1186/s12886-025-04075-y 40312699 PMC12044949

[14] Zhang K Ma X Zhou X Qiu G Zhang C . Machine learning based association between inflammation indicators (NLR, PLR, NPAR, SII, SIRI, and AISI) and all-cause mortality in arthritis patients with hypertension: NHANES 1999–2018. Front Public Health. (2025) 13:1559603. doi: 10.3389/fpubh.2025.1559603 40255373 PMC12007114

[15] Irimie AI Dinescu SN Novac MB Vasile RC Rotaru-Zavaleanu AD Ruscu MA . Monocyte-containing inflammatory indices show stronger association with 30-day mortality than the systemic immune-inflammation index in elderly sepsis: A single-center retrospective observational cohort study. J Clin Med. (2026) 15:4799. doi: 10.3390/jcm15124799 42355967 PMC13301268

[16] Deng R Zhu S Fan B Deng R Zhu S Fan B . Exploring the correlations between six serological inflammatory markers and different stages of type 2 diabetic retinopathy. Sci Rep. (2025) 15:1567. doi: 10.1038/s41598-025-85164-2 39794420 PMC11723939

[17] Wilkinson CP Ferris FL Klein RE Lee PP Agardh CD Davis M . Proposed international clinical diabetic retinopathy and diabetic macular edema disease severity scales. Ophthalmology. (2003) 110:1677–82. doi: 10.1016/S0161-6420(03)00475-5 13129861

[18] Guo L Xiao X . Guideline for the management of diabetes mellitus in the elderly in China (2024 edition). Aging Med. (2024) 7:5–51. doi: 10.1002/agm2.12294 38571669 PMC10985780

[19] Ozawa GY Bearse JMA Adams AJ . Male–female differences in diabetic retinopathy? Curr Eye Res. (2015) 40:234–46. doi: 10.3109/02713683.2014.958500 25545999

[20] Tang H Luo N Zhang X Huang J Yang Q Lin H . Association between biological aging and diabetic retinopathy. Sci Rep. (2024) 14:10123. doi: 10.1038/s41598-024-60913-x 38698194 PMC11065862

[21] Wang S Qin H Zhang Y Yang N Zhao J . The relationship between weight-adjusted-waist index, body mass index and diabetic retinopathy among American adults: a population-based analysis. Sci Rep. (2024) 14:23837. doi: 10.1038/s41598-024-75211-9 39394416 PMC11470029

[22] Lee YJ Kim JJ Kim J Cho DW Won JY . The correlation between waist circumference and the pro-inflammatory adipokines in diabetic retinopathy of type 2 diabetes patients. Int J Mol Sci. (2023) 24:2036. doi: 10.3390/ijms24032036 36768360 PMC9917192

[23] GBD 2019 Blindness and Vision Impairment Collaborators Vision Loss Expert Group of the Global Burden of Disease Study . Trends in prevalence of blindness and distance and near vision impairment over 30 years: an analysis for the Global Burden of Disease Study. Lancet Glob Health. (2021) 9:e130–43. doi: 10.1016/S2214-109X(20)30425-3 33275950 PMC7820390

[24] Teo ZL Tham YC Yu M Chee ML Rim TH Cheung N . Global prevalence of diabetic retinopathy and projection of burden through 2045: systematic review and meta-analysis. Ophthalmology. (2021) 128:1580–91. doi: 10.1016/j.ophtha.2021.04.027 33940045

[25] Dascalu AM Serban D Tanasescu D Vancea G Cristea BM Stana D . The value of White Cell inflammatory biomarkers as potential predictors for Diabetic Retinopathy in type 2 diabetes Mellitus (T2DM). Biomedicines. (2023) 11:2106. doi: 10.3390/biomedicines11082106 37626602 PMC10452280

[26] Choucair K Nebhan C Cortellini A Choucair K Nebhan C Cortellini A . Characterization of age-associated, neutrophil-to-lymphocyte ratio (NLR) and systemic immune-inflammatory index (SII) as biomarkers of inflammation in Geriatric patients with cancer treated with immune checkpoint inhibitors: impact on efficacy and survival. Cancers. (2023) 15:5052. doi: 10.3390/cancers15205052 37894419 PMC10605297

[27] Gu L Xia Z Qing B Wang W Chen H Wang J . Systemic inflammatory response index (SIRI) is associated with all-cause mortality and cardiovascular mortality in population with chronic kidney disease: evidence from NHANES (2001–2018). Front Immunol. (2024) 15:1338025. doi: 10.3389/fimmu.2024.1338025 38558798 PMC10978803

[28] Tuzimek A Dziedzic EA Beck J Kochman W . Correlations between acute coronary syndrome and novel inflammatory markers (systemic immune-inflammation index, systemic inflammation response index, and aggregate index of systemic inflammation) in patients with and without diabetes or prediabetes. J Inflammation Res. (2024) 17:2623–32. doi: 10.2147/JIR.S454117 38707954 PMC11067916

[29] Huang Q Wu H Wo M Ma J Song Y Fei X . Clinical and predictive significance of Plasma Fibrinogen Concentrations combined Monocyte-lymphocyte ratio in patients with Diabetic Retinopathy. Int J Med Sci. (2021) 18:1390. doi: 10.7150/ijms.51533 33628095 PMC7893560

[30] Huang L Li L Wang M Zhang D Song Y . Correlation between ultrawide-field fluorescence contrast results and white blood cell indexes in diabetic retinopathy. BMC Ophthalmol. (2022) 22:231. doi: 10.1186/s12886-022-02442-7 35597915 PMC9123654

[31] Zeng J Chen M Feng Q Wan H Wang J Yang F . The platelet-to-lymphocyte ratio predicts diabetic retinopathy in type 2 diabetes mellitus. Diabetes Metab Syndrome Obesity: Targets Ther. (2022) 15:3617–26. doi: 10.2147/DMSO.S378284 36444389 PMC9700435

[32] Dascalu AM Georgescu A Costea AC Tribus L El Youssoufi A Serban D . Association between neutrophil-to-lymphocyte ratio (NLR) and platelet-to-lymphocyte ratio (PLR) with diabetic retinopathy in type 2 diabetic patients. Cureus. (2023) 15:e48581. doi: 10.7759/cureus.48581 38090430 PMC10711340

[33] Elbeyli A Kurtul BE Ozcan SC Ozarslan Ozcan D . The diagnostic value of systemic immune-inflammation index in diabetic macular oedema. Clin Exp Optometry. (2022) 105:831–5. doi: 10.1080/08164622.2021.1994337 34763621

[34] Wang S Pan X Jia B Chen S . Exploring the correlation between the systemic immune inflammation index (SII), systemic inflammatory response index (SIRI), and type 2 diabetic retinopathy. Diabetes Metab Syndrome Obes. (2023) 16:3827–36. doi: 10.2147/DMSO.S437580 38033457 PMC10683512

[35] Trotta MC Gesualdo C Russo M Lepre CC Petrillo F Vastarella MG . Changes in circulating acylated ghrelin and neutrophil elastase in diabetic retinopathy. Medicina. (2024) 60:118. doi: 10.3390/medicina60010118 38256379 PMC10820226

[36] Tanaka A Imai T Shimabukuro M Nakamura I Matsunaga K Ozaki Y . Effect of canagliflozin on white blood cell counts in patients with type 2 diabetes and heart failure: A subanalysis of the randomized CANDLE trial. J Diabetes Investig. (2022) 13:1990–9. doi: 10.1111/jdi.13899 36114704 PMC9720210

[37] Dihoum A Brown AJ McCrimmon RJ Lang CC Mordi IR . Dapagliflozin, inflammation and left ventricular remodelling in patients with type 2 diabetes and left ventricular hypertrophy. BMC Cardiovasc Disord. (2024) 24:356. doi: 10.1186/s12872-024-04022-7 38997620 PMC11241903

